# Subgroups of self-directed learning ability and their differences in professional identity among nursing undergraduates during the COVID-19 pandemic: a latent profile analysis

**DOI:** 10.1186/s12912-023-01295-9

**Published:** 2023-04-14

**Authors:** Tianji Zhou, Yizhen Yin, Hanyi Zhang, Jie Zhang, Xiaorong Xu, Jingping Zhang

**Affiliations:** 1 Xiangya School of Nursing, Central South University, 172 Tongzipo Road, Changsha, Hunan 410013 China; 2 Xiangya Hospital, Central South University, 87 Xiangya Road, Changsha, Hunan 410008 China; 3 School of Nursing, Hunan University of Chinese Medicine, 300 Xueshi Road, Changsha, Hunan 410208 China; 4 Nursing Department, Hunan Aerospace Hospital, 189 Fenglin Road, Changsha, Hunan 410205 China

**Keywords:** Self-directed learning, Professional identity, Latent profile analysis, Nursing undergraduates, COVID-19, Nursing education

## Abstract

**Background:**

Promoting self-directed learning (SDL) among nursing undergraduates is crucial to meet the new requirements of the healthcare system and to adapt to online learning contexts during the COVID-19 pandemic. Therefore, identifying the classification features of SDL ability and developing targeted interventions are both critical. Professional identity (PI) may contribute to the cultivation of SDL ability, but their relationship remains relatively unknown. This study aimed to explore the subgroups of SDL ability and their differences in PI among nursing undergraduates during the COVID-19 pandemic.

**Methods:**

A total of 2438 nursing undergraduates at four universities in China were enrolled in this cross-sectional study from November 2021 to February 2022. The Self-Directed Learning Scale of Nursing Undergraduates (SLSNU) and the Professional Identity Scale for Nursing Students (PISNS) were administered. A latent profile analysis was performed to explore SDL ability latent profiles. Multinomial logistic regression analysis was conducted to examine the predictors of profile membership, and a one-way analysis of variance was applied to compare the PI scores in each latent profile.

**Results:**

Three latent profiles were identified and labeled ‘low SDL ability’ (n = 749, 30.7%), ‘low initiative of help-seeking’ (n = 1325, 54.4%) and ‘high SDL ability’ (n = 364, 14.9%). Multinomial logistic regression analysis suggested that nursing undergraduates who voluntarily chose a nursing major, had served as a student cadre, and had participated in clinical practicum were less likely to be included in the “low SDL ability” group. The average PI score was statistically different across the three profiles (F = 884.40, p < 0.001).

**Conclusion:**

The SDL ability among nursing undergraduates was divided into three profiles, and results show that promoting PI may effectively foster SDL ability. This study highlights the importance of targeted interventions by considering their distinct SDL ability patterns, especially during the COVID-19 pandemic.

**Supplementary Information:**

The online version contains supplementary material available at 10.1186/s12912-023-01295-9.

## Background

Considering the impact of COVID-19, nursing educators need to transfer most of their coursework online [1, 2], which presents great obstacles for nursing undergraduates as they are required to acquire both theoretical knowledge and operational skills proficiently [3, 4]. Grande et al. [5] concluded that students need self-directed learning (SDL) ability to cope with online learning during the COVID-19 pandemic. The SDL ability of nursing undergraduates includes their self-management ability, information literacy and study cooperation ability [6]. Nursing undergraduates are the backup force of future public health service and nursing teams [7], and enhancing their SDL ability is critical to meet the new requirements for nursing in the new era [8, 9] and to adapt to the challenges posed by COVID-19 to learning methods [10].

Recent studies in China show that nursing undergraduates have low awareness of SDL ability, and many of them even regard it as a burden, which may lead to dropping out of school or even leaving the nursing profession [11]. In addition, nursing education in China is traditionally delivered through face-to-face lectures [3]. Online learning has brought various problems to Chinese nursing students such as poor online literacy and lack of time-management skills [12, 13], all of which are unfavourable to the cultivation of SDL ability. Therefore, investigating the different levels of SDL ability and tailoring interventions are both critical, as it enables nursing educators to support nursing undergraduates with different SDL ability levels and ensure their fitness for future study and work. To date, studies that have examined the SDL ability of nursing undergraduates across different countries [14–17] neither indicated a cut-off for distinguishing different levels nor provided a relevant reference. Furthermore, little is reported on SDL among nursing undergraduates in the context of COVID-19, and some researchers have calculated scale scores to determine the level of SDL ability [5, 18, 19]. In this case, judging by the total score is too simplistic and fails to distinguish subgroups with potentially different characteristics, thereby preventing precise suggestions [20].

Although many studies have explored SDL ability, they have mainly adopted variable-centred analysis methods, which may ignore individual heterogeneity. Moreover, classification and targeted interventions for nursing students with different levels of SDL ability are generally lacking despite their importance during COVID-19. To address these needs, latent profile analysis (LPA) may be a suitable approach. LPA is a person-centred algorithm that identifies subgroups of participants with similar patterns based on variables, thereby dividing participants into different profiles [21].

Another factor we explored was professional identity (PI). PI refers to the nursing undergraduates’ sense of identity with the nature and characteristics of nursing work [22, 23]. Previous studies have shown that PI may contribute to one’s SDL ability [24] and future career choices [25]. As an important factor reflecting nursing undergraduates’ learning enthusiasm and facilitating their future career development [26], the importance of PI is self-evident. Additionally, nursing students had witnessed the important role of nurses during the COVID-19 pandemic, which may contribute to their professional learning and growth [22]. The impact of PI on SDL ability is significantly positive, but the exact effect is uncertain [27]. Therefore, the impact of PI on each profile needs to be investigated given its potential key role in improving SDL ability.

This study employed LPA to (a) explore potentially different profiles in SDL ability, (b) identify the characteristics of each profile, and (c) compare the PIs of latent profiles, thus providing targeted guidance for intervention for enhancing the SDL ability among nursing undergraduates during the COVID-19 pandemic.

## Methods

### Design

A cross-sectional study was conducted, and the STROBE Statement was applied to report the findings of this study (see Appendix 1).

### Participants

Nursing undergraduates at four universities in Hunan Province, China, were recruited as the research participants. The eligibility criteria included: (1) full-time nursing undergraduates; (2) with prior experience in online learning; (3)voluntarily participated in this study and (4) had no cognitive or psychiatric disorders. Suspended, international, and repeat students were excluded as they may not be contacted or communicated with in Chinese.

### Sample size

A minimum sample size of 500 cases is recommended for LPA given that a smaller sample size can introduce problems related to aggregation and identifying small profiles [28]. A total of 2438 participants were included, which met the aforementioned sample size requirements.

### Data collection

An online questionnaire was conducted in this study, which was disseminated through an online data collection website called Questionnaire Star (Wen Juanxing in Chinese) from 5 to 2021 to 10 February 2022. During this time period, the pandemic situation in China was under stable control with about 150 new daily cases of COVID-19 infection. However, in order to avoid a large-scale outbreak of COVID-19 infection among students, nursing undergraduates were required to study online at home or in their school dormitories [29]. In addition, the data was collected before the Chinese winter holidays, so it was possible to ensure that all nursing undergraduates who participated in the survey had an online learning experience.

To ensure its reliability, the survey instrument was piloted among 30 nursing undergraduates in November 2021. The scales were tested to be applicable and the minimum time to answer the questionnaire was 200 s. In the formal investigation, convenience and snowball sampling methods were used to distribute the questionnaire link. An electronic poster that included the purpose, significance and eligibility criteria of this study was also designed. This electronic poster and the questionnaire link were sent together to nursing undergraduates at four universities, and those students who expressed their willingness to participate in this study were encouraged to invite and introduce other potentially eligible students to fill in the questionnaire after securing their permission. The collected questionnaires were then evaluated, and those questionnaires that were answered in less than 200 s were excluded from the analysis as they may not have been filled out carefully.

### Instruments

#### Demographic and study-related characteristics

A self-compiled online questionnaire was used to collect the individual characteristics of the latent profiles of SDL ability, including both demographic data (gender, age, place of residence and grade) and study-related information (voluntary choice of nursing major, student cadre or not, participation in undergraduates’ innovative entrepreneurial training programmes, participation in teachers’ scientific research projects, participation in clinical practicum and career intention).

#### Self-directed learning scale of nursing undergraduates

The Self-Directed Learning Scale of Nursing Undergraduates (SLSNU) developed by Lin and Jiang [6] in China, was used to measure the SDL ability of nursing undergraduates. This 28-item scale includes three subscales: self-management ability (10 items), information literacy (11 items), and study cooperation ability (7 items). SDL ability was gauged using a five-point scale with scores ranging from 1 (complete non-compliance) to 5 (complete compliance). Some of the statements were reversed before calculating the total score. The total score ranges from 28 to 140, with a higher score indicating better SDL ability. The scale was tested for reliability and validity in a valid sample of 4309 cases across China [6], and the Cronbach’s alpha coefficient for the scale ranged from 0.700 to 0.863. The Cronbach’s alpha of SLSNU and three subscales in this study was 0.958, 0.890, 0.914 and 0.835, respectively.

#### Professional identity scale for nursing students

PI was measured using the Professional Identity Scale for Nursing Students (PISNS). It was developed by Hao et al. [30] with 17 items falling into five domains: professional self-image, benefit of retention and risk of turnover, social comparison and self-reflection, independence of career choice, and social modeling. Each item in PISNS is scored from 1 (strongly disagree) to 5 (strongly agree), of which item 12 is scored in reverse. The full score of the scale is 85, with a higher score indicating a higher level of PI. A study of 815 nursing students reported that the Cronbach’s alpha coefficient of this scale was 0.827 [30]. In our study, Cronbach’s alpha coefficient for the total scale was 0.940 and for five domains ranged from 0.850 to 0.952.

### Ethical considerations

This study was approved by the Institutional Review Board at the researchers’ university (Grant Number: E202027). An online informed consent form was presented on the homepage of the online questionnaire. All participants provided their electronic signatures on the consent form and sent it electronically via email or WeChat. The participants were also informed that they would remain anonymous and that all the information they provide would be kept strictly confidential.

### Data analysis

IBM SPSS 26.0 and Mplus 8.3 were used to analyse the data. The tests below were performed using the two-sided test, with p < 0.05 indicating significance.

#### LPA

An exploratory LPA was conducted using Mplus 8.3 to examine the latent profiles of SDL ability among nursing undergraduates. Firstly, five models were estimated by gradually increasing the number of profiles from the initial (1 profile) to the final model (5 profiles) until the fitness metrics reached their optimal levels. Model fitting was performed using a log-likelihood test, and the following metrics were generally adopted to reflect the fitness: the Akaike information criterion (AIC), the Bayesian information criterion (BIC), and the sample size adjusted Bayesian information criterion (aBIC), with a smaller value indicating better model fitness [21]. In LPA, Entropy values are often calculated to evaluate the accuracy of classification ranging from 0 to 1, with higher values preferred. In addition, the p values calculated by the Lo–Mendell–Rubin Test (LMR) and Bootstrap Likelihood Ratio Test (BLRT) are crucial metrics for determining whether the model best suits the data [21]. The p-value < 0.05 indicates that the model fits the data significantly better than the previous model [31].

#### Multinomial logistic regression analysis

After selecting the optimal model, a multinomial logistic regression analysis was performed in SPSS 26.0 to explore the predictors of profile membership.

#### One-way analysis of variance

The difference in the PI scores in each latent profile was obtained via one-way analysis of variance and the Student–Newman–Keuls (SNK) test.

### Common method bias test

The data collection of this study was completed in the same context, and there may be common method biases [32]. All items for SLSNU and PISNS were analyzed using Harman’s single-factor test in SPSS 26.0. If the results show at least two common factors, and the variance explained rate of the first does not exceed 40%, there is no common method bias [33]. The results suggested that five common factors can be extracted and the rate was 31.5%, indicating no obvious common method bias.

## Results

A total of 2516 electronic questionnaires were issued, and 2438 Chinese nursing undergraduates completed the survey, for a drop rate of 3.1%. Most of the participating nursing undergraduates were female (84.9%). These participants were aged from 16 to 24 years, with a mean age of 19.65 years (standard deviation [SD] = 1.37). The proportion of participants in their first to fourth year of schooling all fluctuated around 25%. Additionally, over two-thirds of the participants had served as a student cadre, and nearly three-quarters had participated in clinical practicum (74.9%).

### Latent profiles of self-directed learning ability

Five models were estimated during exploration, whose fit metrics are shown in Table 1. The Log(L), AIC, BIC, and aBIC values in the three-profile model were lower than those of the two-profile model, and the Entropy values of the three-profile model had the highest value (0.957). Meanwhile, the LMR value (p = 0.063) of the four-profile model was not significant, indicating that the three-profile model was better than the four-profile model. Overall, the three-profile model was optimal, and the fit metrics are highlighted in bold in Table 1.

**Table 1:** Fit metrics of each model

Model	k	Log(L)	AIC	BIC	aBIC	Entropy	LMR	BLRT
1 profile	56	-91064.650	182241.300	182566.040	182388.115	-	-	-
2 profiles	85	-80470.659	161111.318	161604.227	161334.162	0.956	0.000	0.000
**3 profiles**	**114**	**-74980.075**	**150188.149**	**150849.228**	**150487.023**	**0.957**	**0.000**	**0.000**
4 profiles	143	-72820.012	145926.025	146755.272	146300.928	0.941	0.063	0.000
5 profiles	172	-71098.277	142540.554	143537.970	142991.486	0.952	0.357	0.000

Abbreviations: k, Number of free parameters; Log(L), Log-likelihood value; AIC, Akaike information criterion; BIC, Bayesian information criteria; aBIC, adjusted Bayesian information criteria; LMR, Lo–Mendell–Rubin Test; BLRT, Bootstrap Likelihood Ratio Test

**Fig. 1:**
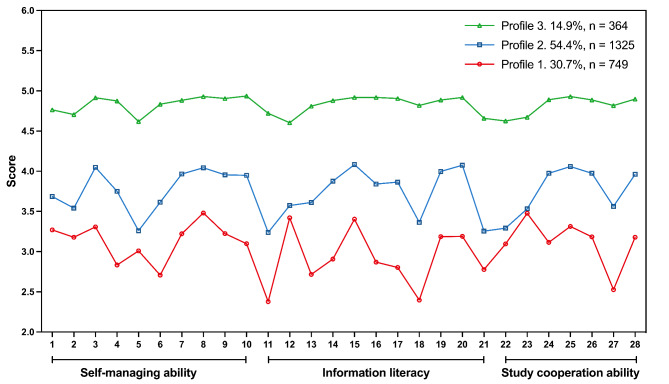
Latent profiles of self-directed learning ability among nursing undergraduates For data analysis, the original scale items were rearranged according to the items contained in each dimension, with items (1–10) for self-managing ability, items (11–21) for information literacy, and items (22–28) for study cooperation ability

The scores of three profiles on 28 items of three dimensions are shown in Fig. 1. Profile 1 was named the ‘low SDL ability’ group, accounting for 14.9% (n = 364) of all participants. It was notable that undergraduates in this profile reported the lowest score for all items. Profile 2 was named the ‘low initiative of help-seeking’ group and accounted for 54.4% (n = 1325). The scores of all items in Profile2 were relatively higher than that of Profile 1, except for item 12 (‘I am not familiar with nursing websites’), item 22 (‘I will not ask the teacher for advice after class though having questions’), and item 23 (‘I do not have much contact with teachers except in class’). Items 12, 22, and 23 were all scored in reverse, with lower scores indicating greater conformity to the statement. In other words, nursing undergraduates in Profile 2 had a lower initiative to seek help from their teachers or external resources such as websites. Finally, nursing undergraduates in Profile 3 reported the highest scores for all items and accounted for the remaining 14.9% (n = 364) of the sample. Therefore, ‘high SDL ability’ was named for this subgroup.

### Demographic and study-related characteristics of each profile

The demographic and study-related characteristics of the participants are presented in Table 2, and the age was divided into three groups as nursing undergraduates are generally aged between 19 and 21 years [9]. The ‘low SDL ability’ group accounted for the smallest percentage of residing in urban areas (37.8% vs. 41.8% vs. 46.7%) and voluntarily choosing to major in nursing (31.6% vs. 41.8% vs. 44.2%). The ‘high SDL ability’ group accounted for the largest proportion of undergraduates who had taken part in innovative entrepreneurial training programs (41.5% vs. 35.1% vs. 37.1%) and teachers’ scientific research projects (21.2% vs.11.5% vs. 14.3%).

**Table 2:** Demographic and study-related features by latent profile membership

	Overall (N = 2438) n (%)	Profile 1 (n = 749) n (%)	Profile 2 (n = 1325) n (%)	Profile 3 (n = 364) n (%)
Gender				
Male	368 (15.1)	118 (15.8)	173 (13.1)	77 (21.2)
Female	2070 (84.9)	631 (84.2)	1152 (86.9)	287 (78.8)
Age				
≤18	564 (23.1)	177 (23.6)	300 (22.7)	87 (23.9)
19 ~ 21	1676 (68.8)	518 (69.2)	908 (68.5)	250 (68.7)
≥22	198 (8.1)	54 (7.2)	117 (8.8)	27 (7.4)
Place of residence				
Rural	1431 (58.7)	466 (62.2)	771 (58.2)	194 (53.3)
Urban	1007 (41.3)	283 (37.8)	554 (41.8)	170 (46.7)
Grade				
First-year student	657 (26.9)	208 (27.8)	342 (25.8)	107 (29.4)
Second-year student	599 (24.6)	179 (23.9)	327 (24.7)	93 (25.5)
Third-year student	661 (27.1)	228 (30.4)	349 (26.3)	84 (23.1)
Fourth-year student	521 (21.4)	134 (17.9)	307 (23.2)	80 (22.0)
Voluntary choice of the nursing major				
Yes	952 (39.0)	237 (31.6)	554 (41.8)	161 (44.2)
No	1486 (61.0)	512 (68.4)	771 (58.2)	203 (55.8)
Student cadre or not				
Yes	1646 (67.5)	450 (60.1)	929 (70.1)	267 (73.4)
No	792 (32.5)	299 (39.9)	396 (29.9)	97 (26.6)
Participation in undergraduates’ innovative entrepreneurial training programs				
Yes	905 (37.1)	263 (35.1)	491 (37.1)	151 (41.5)
No	1533 (62.9)	486 (64.9)	834 (62.9)	213 (58.5)
Participation in teachers’ scientific research projects				
Yes	352 (14.4)	86 (11.5)	189 (14.3)	77 (21.2)
No	2086 (85.6)	663 (88.5)	1136 (85.7)	287 (78.8)
Participation in clinical practicum				
Yes	1827 (74.9)	495 (66.1)	1045 (78.9)	287 (78.8)
No	611 (25.1)	254 (33.9)	280 (21.1)	77 (21.2)
Career intention				
Clinical nursing work	1070 (43.9)	303 (40.4)	604(45.6)	163 (44.8)
Further education	897 (36.8)	248 (33.1)	517 (39.0)	132 (36.3)
Non-nursing careers	311 (12.7)	136 (18.2)	130 (9.8)	45 (12.3)
Others	160 (6.6)	62 (8.3)	74 (5.6)	24 (6.6)

Profile 1: Low self-directed learning ability profile, Profile 2: Low initiative of help-seeking profile, Profile 3: High self-directed learning ability profile

### Predictor of latent profile membership

To identify the predictors of profile membership, a multinomial logistic regression was conducted with the ‘low SDL ability’ group as the reference. The Predictors are highlighted in bold in Table 3. Nursing undergraduates who were in their fourth-year study (OR = 1.472, p = 0.046) and chose to pursue further studies (OR = 1.494, p = 0.04) were more likely to be in the ‘low initiative of help-seeking’ group. Whereas those residing in urban areas (OR = 1.406, p = 0.01) and having participated in teachers’ scientific research projects (OR = 1.738, p = 0.005) were more likely to be in the ‘high SDL ability’ group. Compared to individuals in the ‘low SDL ability’ group, students who voluntarily chose the nursing major, had served as a cadre and had taken part in clinical practicum were more likely to be in the second and third profiles.

**Table 3:** Predictor of latent profile membership

	B	SE	OR	95% confidence interval	p
Profile 2: Low initiative of help-seeking (vs. Profile 1: Low self-directed learning ability)					
Gender: male, ref.: female	-0.218	0.133	0.804	0.619–1.044	0.102
Age: 19 ~ 21, ref.: ≤18	-0.051	0.157	0.950	0.699–1.293	0.745
Age: ≥22, ref.: ≤18	0.004	0.251	1.005	0.615–1.643	0.984
Place of residence: urban, ref.: rural	0.154	0.097	1.166	0.964–1.411	0.114
Grade: second-year, ref.: first-year	0.251	0.159	1.285	0.941–1.754	0.115
Grade: third-year, ref.: first-year	0.035	0.173	1.035	0.738–1.453	0.841
**Grade: fourth-year, ref.: first-year**	**0.387**	**0.194**	**1.472**	**1.007–2.153**	**0.046**
**Voluntary choice of the nursing major: Yes, ref.: No**	**0.383**	**0.102**	**1.466**	**1.202–1.789**	**0.000**
**Student cadre: Yes, ref.: No**	**0.296**	**0.102**	**1.342**	**1.101–1.641**	**0.004**
Participation in undergraduates’ innovative entrepreneurial training programs: Yes, ref.: No	-0.120	0.111	0.887	0.713–1.103	0.280
Participation in teachers’ scientific research projects: Yes, ref.: No	0.072	0.155	1.075	0.794–1.455	0.640
**Participation in clinical practicum: Yes, ref.: No**	**0.548**	**0.108**	**1.730**	**1.401–2.137**	**0.000**
Career intention: clinical nursing work, ref.: others	0.319	0.192	1.376	0.944–2.006	0.096
**Career intention: further education, ref.: others**	**0.402**	**0.195**	**1.494**	**1.019–2.191**	**0.040**
Career intention: non-nursing careers, ref.: others	-0.258	0.216	0.772	0.506–1.180	0.233
Profile 3: High self-directed learning ability (vs. Profile 1: Low self-directed learning ability)					
Gender: male, ref.: female	0.309	0.168	1.363	0.981–1.892	0.065
Age: 19 ~ 21, ref.: ≤18	0.097	0.213	1.102	0.726–1.671	0.649
Age: ≥22, ref.: ≤18	-0.002	0.350	0.998	0.503–1.980	0.995
**Place of residence: urban, ref.: rural**	**0.341**	**0.133**	**1.406**	**1.083–1.826**	**0.010**
Grade: second-year, ref.: first-year	0.057	0.215	1.059	0.695–1.615	0.789
Grade: third-year, ref.: first-year	-0.366	0.239	0.693	0.434–1.107	0.125
Grade: fourth-year, ref.: first-year	0.047	0.261	1.048	0.628–1.749	0.858
**Voluntary choice of the nursing major: Yes, ref.: No**	**0.477**	**0.139**	**1.611**	**1.228–2.114**	**0.001**
**Student cadre: Yes, ref.: No**	**0.382**	**0.147**	**1.465**	**1.097–1.955**	**0.010**
Participation in undergraduates’ innovative entrepreneurial training programs: Yes, ref.: No	0.015	0.155	1.015	0.749–1.375	0.925
**Participation in teachers’ scientific research projects: Yes, ref.: No**	**0.553**	**0.195**	**1.738**	**1.186–2.546**	**0.005**
**Participation in clinical practicum: Yes, ref.: No**	**0.474**	**0.157**	**1.606**	**1.181–2.182**	**0.002**
Career intention: clinical nursing work, ref.: others	0.122	0.268	1.130	0.668–1.911	0.648
Career intention: further education, ref.: others	0.079	0.273	1.082	0.634–1.846	0.773
Career intention: non-nursing careers, ref.: others	-0.214	0.302	0.808	0.447–1.460	0.479

### PI with latent profile membership

Analysis of variance was conducted to explore the differences in the PI of the three profiles (Table 4). The mean scores of the PISNS of nursing undergraduates in Profiles 1, 2 and 3 were 54.37 (SD = 10.22), 64.65 (SD = 8.27) and 77.79 (SD = 7.74), respectively. As shown in Table 4, the scores of PISNS and the five dimensions statistically differed across the three profiles (p < 0.001). Moreover, the SNK test revealed that the mean score of the ‘high SDL ability’ group was significantly higher than that of the ‘low SDL ability’ group and the ‘low initiative of help-seeking’ group, whereas the figure for the ‘low SDL ability’ group was the lowest.

**Table 4:** Professional identity difference of three profiles

	Profile 1 (n = 749) M ± SD	Profile 2 (n = 1325) M ± SD	Profile 3 (n = 364) M ± SD	F	p	SNK
Professional identity	54.37 ± 10.22	64.65 ± 8.27	77.79 ± 7.74	884.40	0.000	3 > 2 > 1
Professional self-image	18.48 ± 4.53	22.65 ± 4.03	28.45 ± 3.69	724.53	0.000	3 > 2 > 1
Benefit of retention and risk of turnover	11.87 ± 2.97	14.67 ± 2.79	18.83 ± 2.65	752.91	0.000	3 > 2 > 1
Social comparison and self-reflection	10.45 ± 2.12	12.18 ± 1.47	14.56 ± 1.31	746.59	0.000	3 > 2 > 1
Independence of career choice	6.79 ± 1.28	6.97 ± 1.35	6.30 ± 1.09	38.57	0.000	3 > 2 > 1
Social modeling	6.78 ± 1.79	8.17 ± 1.26	9.64 ± 1.07	529.00	0.000	3 > 2 > 1

Profile 1: Low self-directed learning ability profile, Profile 2: Low initiative of help-seeking profile, Profile 3: High self-directed learning ability profile, M: Mean, SD: standard deviation, SNK: Student–Newman–Keuls.

## Discussion

### Latent profiles of SDL

By taking a person-centred approach to analyse the SDL ability of nursing undergraduates during the COVID-19 pandemic, this study aimed to highlight the differences in their SDL ability and to guide further research on tailored SDL ability improvement according to latent profiles. To the best of the authors’ knowledge, this study is the first to use LPA to identify the latent profiles of SDL ability among nursing undergraduates, hence complementing previous studies that treat nursing undergraduates as a homogeneous whole. This study also enriches the exploration of the SDL ability of nursing undergraduates in the context of COVID-19. Therefore, this study helps to develop targeted intervention measures according to the characteristics of the different profiles of nursing undergraduates.

The findings of this study revealed the distinct categorical features of the SDL ability among nursing undergraduates during the COVID-19 pandemic. Based on the score responses for each item, three profiles were identified, namely, the ‘low SDL ability’, ‘low initiative of help-seeking’ and ‘high SDL ability’ groups. This classification reflects the heterogeneity of nursing undergraduates in each latent profile and can be used as a reference for comparison in the future.

The ‘low SDL ability’ group consisted of 30.7% of the sample. Nursing undergraduates in this profile had poor awareness of SDL and relatively weak self-monitoring skills. Their low SDL ability can be ascribed to several factors. Firstly, the current nursing curriculum system still emphasizes a teacher-centred approach, which focuses on summative assessments and remembering facts [34]. Moreover, nursing undergraduates may lack confidence in their own abilities and therefore need to be challenged to actively participate in planning and designing their learning process, which is consistent with the findings of Senyuva and Kaya [35]. Secondly, nursing educators undertake the additional task of developing online educational content at short notice during the COVID-19 pandemic [36], and some courses may be of low quality and not interesting enough to keep students engaged. Thirdly, the lack of self-management skills is undoubtedly the biggest barrier to SDL [37]. One distinguishing feature of online learning is that students experience a higher level of autonomy in their learning [3]. In this case, nursing undergraduates are principally responsible for their own learning without the supervision of teachers, and some of them do not know how to manage their time to study [38]. Therefore, for this profile, interventions should focus on stimulating learning passion and establishing self-efficacy of nursing undergraduates.

The ‘low initiative of help-seeking’ group, comprising 35.7%, had relatively low scores on items 12, 22, and 23. Judging from these items, nursing undergraduates in this group were less likely to seek help from their teachers and had poor ability to access nursing information online. Academic help-seeking is an important metacognitive skill in education and refers to engagement in support that improves one’s academic performance [39]. The results for this profile revealed that nursing undergraduates rarely communicate with teachers after class and do not ask for help though having questions. This finding may be related to the limited number of nursing faculty and the limited contact between teachers and students [40]. Some teachers have to balance their administration, research and clinical practice, and their teaching process is mostly divided into Sect.  [41]. Moreover, online learning during COVID-19 may have reduced the opportunities for the participants to communicate with teachers [5]. For information literacy, nursing undergraduates in this group had relatively weak ability to access online information. In a systematic review [42], nursing undergraduates reported that the main barriers related to finding information on the Internet included lack of time, insufficient retrieval skills, and poor awareness of the library as a reliable and efficient tool. The closure of campus infrastructure and libraries during COVID-19 also exacerbated their difficulty in seeking information help. In addition, some nursing undergraduates believe that they need to actively obtain information only for scientific research, while they are mainly engaged in clinical nursing and have nothing to do with nursing research [43]. Above all, the undergraduates in this profile had poor initiative to seek help both offline and online. Therefore, nursing educators should broaden the information acquisition channels of their students and establish positive relationships with them.

The ‘high SDL ability’ group, which accounted for the remaining 14.9% of the sample, had the highest level of SLSNU items. These undergraduates had greater self-management, information acquisition and collaborative learning abilities. The better SDL ability of nursing undergraduates in this group is mainly attributed to the promotion of modern course platforms during COVID-19, hence making learning styles flexible and access to knowledge portable [44]. In this case, students have more control over their own learning, including personal learning strategies and setup [45]. In addition, nursing undergraduates in this group had relatively good study cooperation ability and information literacy. On the one hand, the development of certain courses, such as literature retrieval and nursing research methods, enhanced their ability to process and integrate information independently and identify effective learning resources [46]. On the other hand, new forms of nursing education, such as flipped classrooms and group discussions, have emerged in recent years. Most nursing undergraduates can communicate in a timely and effective manner [37, 44], thereby increasing their interactivity in learning and cultivating their study cooperation ability. Whilst these undergraduates had satisfactory SDL ability, it is not optimal, given that online and hybrid learning was treated as the new normal [18]. Therefore, the interventions for these undergraduates should focus on encouraging their achievements and continuously exploring innovative teaching methods. Nursing educators are suggested to explore innovative strategies, such as interactive online simulation programmes and interprofessional telehealth education [18], which may further improve their SDL ability and build their critical thinking and problem-solving skills.

### Demographic and study-related characteristics of each profile

The demographic predictors of profile membership include grade and place of residence. For example, nursing undergraduates in their fourth-year study were more likely to be in the ‘low initiative of help-seeking’ group. In China, most senior nursing undergraduates have already enrolled in clinical practice [47]. In this case, they believe that skill operation is at the core of clinical work and generally do not learn new knowledge through websites [43]. In addition, the senior year is a period when students shift from school to clinical practice, with less academic pressure and less contact with teachers given the lack of rigorous exams. However, these findings contradict those of other studies [9, 48, 49]. Therefore, a causal relationship cannot be deduced between senior students and the ‘low help-seeking initiative’ group without theoretical and empirical evidence. Meanwhile, those nursing undergraduates who reside in urban areas were more likely to be in the ‘high SDL ability’ group as they have richer learning resources and better learning environments [50].

The study-related predictors of profile membership in this study include voluntary choice of the nursing major, student cadre, and participation in clinical practicum. Those students who voluntarily choose to major in nursing were less likely to be in the ‘low SDL ability’ group. In the learning process, attitudes and affection for the profession, as non-intellectual factors, are extremely critical in stimulating interest in learning and improving learning performance [51], which determine the SDL ability. Additionally, those nursing undergraduates who have served as cadres were more likely to be in the second and third groups, which is consistent with the findings of Zhou et al [52]. Compared with their counterparts, student cadres possess better communication and problem-solving skills and higher emotional and intellectual quotient [53]. Furthermore, a significant difference was also observed in terms of their participation in clinical practicum, as early clinical practicum could urge nursing undergraduates’ motivation in SDL [54]. The clinical learning environment differs from the theoretical teaching environment in schools, with various real-time feedback enabling students to identify their deficiencies and promote their SDL ability.

### PI of the three profiles

The average score of PI of the ‘high SDL ability’ group was notably higher than those of the other two groups, and the higher PI indicates that nursing undergraduates are more attentive to their study of professional courses and have stronger SDL ability. Some studies have highlighted a positive relationship between nursing students’ PI and SDL ability [27, 55]. Specifically, students with a high level of PI accept the nursing profession deelply and make positive perceptions and evaluations, therefore, they actively study hard to achieve the goal of success in the filed [56]. In addition, out of intrinsic interest, they actively learn and explore professional knowledge and skills, and are willing to choose and solve complex problems, which improves their own quality and SDL ability [57].

The PI of nursing undergraduates increased during COVID-19 due to the public support and recognition of nurses [22]. The pandemic created a vivid classroom for them to gain an in-depth and comprehensive understanding of the nursing profession, thereby increasing their awareness of their own value and social responsibility [22]. Therefore, nursing educators should strengthen the professional values of nursing undergraduates through various methods, such as international nurses day and nursing role models [56]. Instructing students to develop a sense of professional value and mission can stimulate their intrinsic learning motivation and cultivate their SDL ability. Above all, promoting PI may be an effective method of fostering one’s SDL ability.

### Implications

When developing targeted interventions for SDL ability among nursing undergraduates, nursing educators should pay attention to each profile’s characteristics as shown in the LPA results. For the ‘low SDL ability’ group, the focus should be on the transformation of traditional teaching methods. Nursing educators should create a student-centred learning environment, and develop teaching strategies, such as problem-based learning and clinical scenario simulation exercises, which may increase the students’ awareness of SDL ability [58]. As for the ‘low initiative of help-seeking’ group, developing positive teacher-student relationships and broadening access to information are critical. With the emergence of online learning, teachers should make full use of online platforms to increase their video or voice interaction with students [18]. Schools should also introduce information-based education into nursing courses and provide information retrieval courses to enhance students’ information literacy. Those undergraduates in the ‘high SDL ability’ group should be encouraged to enhance their sense of achievement. Nursing educators should also explore innovative strategies to promote the comprehensive development of nursing undergraduates’ SDL ability, critical thinking disposition and problem-solving skills [8]. Early clinical practicums, career planning education and promotion of nursing role models during COVID-19 can also be advocated to cultivate the PI and reinforce the SDL ability of nursing undergraduates [52].

### Limitations

There are some limitations to this study. Firstly, the nursing undergraduates were recruited via convenience and snowball sampling from a single region of China, hence limiting the representativeness of the sample. Secondly, as a cross-sectional study, the findings of this work cannot be used to determine cause and effect, so the causation between SDL ability and PI cannot be identified. Further longitudinal studies should be conducted to track the trajectory of SDL ability over time. Thirdly, given that the majority of the participants were women, gender bias may not be completely avoided. Therefore, as the number of male students taking nursing curricula increases [9], future studies should recruit more male nursing undergraduates.

## Conclusions

This study demonstrated the obvious classification characteristics of SDL ability among nursing undergraduates during the COVID-19 pandemic and proposed a three-profile model involving the ‘low SDL ability’ group, ‘low initiative of help-seeking’ group and ‘high SDL ability’ group. From the person-centred perspective, targeted interventions should be formulated based on the demographic and study-related characteristics of each profile. Moreover, promoting PI can be an effective approach to fostering SDL ability. In conclusion, enhancing SDL ability is crucial for nursing undergraduates to meet the new requirements of the healthcare system and to adapt to new forms of online learning due to the COVID-19 pandemic.

## Electronic supplementary material

Below is the link to the electronic supplementary material.

Supplementary Material 1

## Data Availability

The datasets generated and/or analysed during the current study are available on the Open Science Framework (OSF) websites (https://osf.io/tydnh), registration DOI: 10.17605/OSF.IO/PUM7R.

## Abbreviations

COVID-19: Coronavirus Disease 2019
SDL: Self-directed Learning
PI: Professional Identity
SLSNU: Self-Directed Learning Scale of Nursing Undergraduates
PISNS: Professional Identity Scale for Nursing Students
LPA: Latent Profile Analysis
Log(L): Log-likelihood value
AIC: Akaike information criterion
BIC: Bayesian information criterion
aBIC: Sample size adjusted Bayesian information criterion
LMR: Lo–Mendell–Rubin Test
BLRT: Bootstrap Likelihood Ratio Test
SNK: Student–Newman–Keuls
SD: Standard deviation
